# Ultrasound Assessment of Diaphragm in Patients with Chronic Obstructive Pulmonary Disease, Interstitial Lung Disease and Healthy Volunteers: An Observational Study

**DOI:** 10.31729/jnma.9186

**Published:** 2025-08-31

**Authors:** Laxman Thakur, Prajowl Shrestha, Kamal Raj Thapa, Deepa Kumari Shrestha, Avatar Verma, Mukti Nath Khanal, Ashesh Dhungana

**Affiliations:** 1 Pulmonary, Critical Care and Sleep Medicine, National Academy of Medical Sciences, Bir Hospital, Kathmandu, Nepal; 2 Bagmati Sewa Samaj Nepal, Kathamdnu Nepal

**Keywords:** *chronic obstructive pulmonary disease*, *diaphragm*, *interstitial lung disease*, *ultrasound*, *volunteers*

## Abstract

**Introduction:**

The diaphragm dysfunction is being increasingly recognized in chronic respiratory diseases. Ultrasonography offers a simple, non-invasive method for assessing diaphragmatic structure and function. This study aimed to evaluate and compare diaphragm thickness and excursion in patients with Chronic Obstructive Pulmonary Disease, Interstitial Lung Disease and healthy volunteers.

**Methods:**

A cross-sectional observational study was conducted among patients with Chronic Obstructive Pulmonary Disease, Interstitial Lung Disease and healthy controls. Diaphragm ultrasonography was performed to measure thickness at tidal expiration and tidal inspiration, maximal inspiration , thickening fraction, diaphragmatic excursion during quiet breathing, and maximal inspiration. Mean values were calculated and compared across groups. Correlations between mMRC score and FEV1 and FVC were also assessed.

**Results:**

A total of 75 participants were included: 25 Chronic Obstructive Pulmonary Disease patients, Interstitial Lung Disease patients and healthy volunteers each. All the parameters on both sides were comparable. Diaphragm thickness during tidal breathing were similar across groups. Diaphragm thickness during maximal inspiration and thickening fraction were lowest in Chronic Obstructive Pulmonary Disease, intermediate in Interstitial Lung Disease, and highest in healthy controls. Diaphragmatic excursion was reduced in Chronic Obstructive Pulmonary Disease compared with Interstitial Lung Disease and controls, with similar trends in maximal inspiration. Dyspnea severity negatively correlated with FEV1 and FVC in both Chronic Obstructive Pulmonary Disease and Interstitial Lung Disease patients.

**Conclusions:**

Chronic Obstructive Pulmonary Disease patients exhibited the greatest impairment in diaphragmatic contractility and excursion, followed by Interstitial Lung Disease, taking healthy volunteers as reference. Diaphragm ultrasonography provides useful physiological insights and may serve as an adjunctive tool in assessing respiratory muscle function in chronic lung disease.

## INTRODUCTION

Chronic obstructive pulmonary disease (COPD) and interstitial lung diseases (ILD) are major chronic respiratory conditions with rising global burden. COPD is the fourth leading cause of death worldwide.^1,2^ The reported prevalence of ILDs is 33.6 to 98 per 100,000 population. ^3,4^ The diaphragm plays a central role in respiration, and its dysfunction is being increasingly recognized in chronic respiratory diseases.^5-7^ The proposed pathophysiological mechanisms for diaphragmatic dysfunction are physical deconditioning, pro-inflammatory states, hypoxia, malnutrition, sarcopenia and corticosteroid use.^8, 9^

Ultrasound is a rapid, simple, non-invasive, easy-to-perform, repeatable, inexpensive, and radiation-free examination technique used to evaluate the anatomy and function of diaphragm. Diaphragm thickness (DT) and diaphragm excursion (DE) are used to see the anatomical and functional status of the diaphragm in an objective way. This study aims to assess diaphragm function in healthy volunteers and patients with COPD and ILD.

## METHODS

This was a cross-sectional observational study conducted at National Academy of Medical Sciences (NAMS), Bir Hospital from May 2024 to April 2025. Ethical approval was taken from the Institute Review Board (Reference no. 48/2081/82). Diaphragm function of 25 patients with COPD, 25 patients with ILD and 25 healthy volunteers were assessed. Patient with Pulmonary Function Test (PFT) with ratio of Forced Expiratory Volume in one second (FEV1) to Forced Vital Capacity (FVC) less than Lower Limit of Normal (LLN) with FEV1 Z score < -2.5 were enrolled as COPD.^10,11^ Similarly, patients having unequivocal evidence of ILD in Computed Tomography chest with FVC Z score < -2.5 were enrolled as ILD.^10,11^ Individuals with no history of smoking, no respiratory symptoms or chronic respiratory diseases and having normal spirometry were selected as healthy volunteers. Patients with concurrent respiratory diseases, neuromuscular diseases, chest wall deformity, unilateral diaphragm weakness and acute exacerbation were excluded from the study. Spirometry was performed in PFT lab with the use of Easy on - PC Spirometer [GTIN (01): 7640142190301, MFD: 2021-0909]). The presence of dyspnea was assessed by modified Medical Research council(mMRC) Questionnaire.^12^

Assessment of diaphragm function was done measuring Diaphragm Thickness (DT) [during tidal expiration (DTe), tidal inspiration (DTi) & maximal inspiration (DTmax)], Thickness fraction (TF) and Diaphragm excursion [during tidal inspiration (DE) and during maximal inspiration (DEmax)]. All diaphragm USG examination were performed with USG machine model Fujifilm Sonosite Edge II total Q5JK2. The exams were conducted by physicians trained in performing diaphragm ultrasound. Patients were asked to lie in a supine position, and assessment of both the hemidiaphragm were done. Measurement of the DT was done by linear ultrasound probe HFL38(6-13 MHz) by B-mode and diaphragm excursion (DE) by convex ultrasound probe C60 (2-5 MHz) by M-mode. Three measurements were taken for each parameter, and the mean value was used for the analysis. The unit of measurement was centimeter (cm).

Diaphragm thickness was measured in the zone of apposition in the bilateral hemithorax between the anterior axillary line and the mid-axillary line between the eighth and eleventh intercostal spaces in longitudinal intercostal view. Diaphragm thickness was defined as the distance between the diaphragmatic pleura and the peritoneal membrane. Measurements at the end of expiration and at end of the inspiration both during quiet tidal breathing and maximal deep breathing were taken^13^

The Thickening Fraction (TF) was calculated with the DT value. [(DT at end-inspiration) - (DT at end-expiration)]/ (DT at endexpiration) × 100. ^13,14^

Diaphragmatic excursion (DE) was measured at the anterior subcostal margin with the use of anterior subcostal view. Convex probe was placed below the costal margin between the midclavicular line and the anterior axillary line. The right and left hemi-diaphragms were evaluated via the liver and spleen windows respectively. The hemi-diaphragm was visible as thick, curving hyperechoic line. The diaphragm excursion which is the diaphragm inspiratory amplitude measured in M-mode, was measured during quiet tidal breathing (DE) and maximal deep breathing (DEmax).^13^

Categorical variables were presented as absolute number and percentages and continuous variables as mean and standard deviation.

## RESULTS

A total of 75 individuals were enrolled in our study, 25 each in COPD, ILD and Healthy volunteers’ group. The mean age of the participants enrolled in COPD, ILD and healthy volunteers’ group were 65.4± 8.17 years, 57.12±15.03 years and 42.4±10.23 years respectively (Table 1).

**Table 1:** Demographic and clinical characteristics of study participants (n=75).

Parameter	COPD (n=25) n(%)	ILD (n=25) n(%)	Healthy Volunteers (n=25) n(%)
Age (years)	65.4±8.17	57.12±15.03	42.4±10.23
Gender			
Male	16(64)	9(36)	13(48)
Female	9(36)	16(64)	12(52)
mMRC Dyspnea Scale			
Grade 0	3(12)	2(8)	0
Grade 1	9(36)	10(40)	0
Grade 2	7(28)	8(32)	0
Grade 3	3(12)	3(12)	0
Grade 4	2(8)	2(8)	0
Spirometry			
FEV_1_ (L)	1.07±0.29	1.28±0.37	2.15±0.40
FVC (L)	2.15±0.60	1.44±0.37	2.53±0.48

mMRC=modified Medical Research Council, FEVi: Forced Expiratory Volume in one second, FVC=Forced Vital Capacity.

**Table 2:** Age adjusted Means and pairwise comparisons of diaphragm parameters among COPD patients, ILD patients and Healthy Volunteers

Parameters	COPD (Mean±SD)	ILD (Mean±SD)	Healthy volunteers (Mean±SD)
DTe(Right)	0.12±0.02	0.12±0.02	0.11±0.02
DTe(Left)	0.12±0.015	0.11±0.02	0.11±0.02
DTi(Right)	0.16±0.03	0.16±0.03	0.16±0.04
DTi(Left)	0.15±0.03	0.16±0.02	0.16±0.03
DTimax(Right)	0.27±0.04	0.31±0.04	0.32±0.04
DTimax(Left)	0.27±0.04	0.29±0.04	0.32±0.04
TF(Right)	27.78±8.18	39.93±7.29	49.64±8.55
TF(Left)	30.87±14.61	42.18±13.02	46.49±15.26
DE(Right)	1.44±0.45	1.69±0.41	1.82±0.47
DE(Left)	1.40±0.40	1.61±0.36	1.80±0.42
DEmax(Right)	4.02±0.96	4.34±0.85	5.45±1.0
DEmax(Left)	3.93±0.86	4.19±0.77	5.37±0.90

SD=Standard Deviation; DTe=Diaphragmatic thickness at end-expiration; DTi=Diaphragmatic thickness at endinspiration; DTimax=Maximal inspiratory diaphragmatic thickness; TF=Thickening fraction; DE=Diaphragmatic excursion during quiet breathing; DEmax=Diaphragmatic excursion during deep breathing; COPD= Chronic Obstructive Pulmonary Disease; ILD=Interstitial Lung Disease

Baseline diaphragm thickness at end-expiration (DTe) and endinspiration (DTi) during tidal breathing was comparable across the three groups, with no clinically relevant differences observed. However, maximal diaphragm thickness during inspiration (DTimax) was lowest in COPD patients (right: 0.27±0.04 cm; left: 0.26±0.04 cm), intermediate in ILD patients (right: 0.31±0.035 cm; left: 0.29±0.035 cm), and highest in healthy volunteers (right: 0.32±0.04 cm; left: 0.32 ± 0.04 cm). Thickening fraction (TF) showed a similar trend, with low values in COPD (right: 27.8 ± 8.2%; left: 30.9± 14.6%) compared to ILD (right: 39.9 ± 7.3%; left: 42.2±13.0%) and healthy controls (right: 49.6±8.6%; left: 46.5 ± 15.3%). Diaphragmatic excursion (DE) during quiet breathing was also low in COPD (right: 1.44±0.45 cm; left: 1.40±0.40 cm) compared with ILD (right: 1.69 ± 0.41 cm; left: 1.61 ± 0.36 cm) and healthy volunteers (right: 1.82 ± 0.47 cm; left: 1.80 ± 0.42 cm). Similarly, maximum diaphragmatic excursion (DEmax) was lowest in COPD (right: 4.02 ± 0.96 cm; left: 3.93 ± 0.86 cm), higher in ILD (right: 4.34 ± 0.85 cm; left: 4.19 ± 0.77 cm), and highest in healthy participants (right: 5.45 ± 1.0 cm; left: 5.37 ± 0.90 cm).

The mMRC dyspnea grade negatively correlated with FEV1 (r = -0.70) and FVC (r = -0.55) in COPD patients. The study showed significant negative correlation between mMRC and FEV1 (r = -0.75) and FVC (r = -0.76) in ILD patients as well (Table 3).

**Table 3:** Correlation between dyspnea severity (mMRC grade) and pulmonary function parameters in COPD and ILD patients (n=75).

	COPD		ILD	
	FEV1	FVC	FEV1	FVC
mMRC	r = -0.70	r = -0.55	r = -0.75	r = -0.76

mMRC=Modified Medical Research Council; COPD=Chronic Obstructive Pulmonary Disease; ILD=Interstitial Lung Disease; FEV1=Forced Expiratory Volume in One Second; FVC=Forced Vital Capacity

## DISCUSSION

Our study evaluated diaphragmatic function using ultrasonography in patients with COPD, ILD, and healthy volunteers. We found that diaphragm thickness during tidal breathing was preserved across all groups, a result that is in agreement with previous studies.^15,16^ This suggests that baseline thickness measured during quiet breathing may not serve as a reliable or sensitive indicator of diaphragmatic dysfunction. Nonetheless, there is some inconsistency in the literature. A systematic review reported heterogeneous findings where nine studies demonstrated significantly reduced diaphragm thickness in COPD patients compared with controls, one study found increased thickness, while three reported no significant differences.^17^ Such variability may reflect differences in methodology, disease severity, and patient characteristics.

In our study participants, COPD patients exhibited a lower diaphragmatic thickness (0.27±0.04 cm in both right and left) during maximum inspiration (DTimax) compared to both ILD patients (0.31±0.04 cm in right and 0.29±0.04 cm in left) and healthy individuals (0.32±0.04 cm in both right and left). This finding reflects impaired contractility of the diaphragm in COPD, most likely due to the deleterious effects of chronic hyperinflation. In contrast, ILD patients showed a reduction in inspiratory thickness compared with healthy controls, but values were relatively preserved when compared to COPD patients. This pattern indicates that restrictive lung physiology, while impairing diaphragm motion due to fibrosis and reduced chest wall compliance, tends to spare contractile properties to a greater extent than obstructive physiology. Importantly, the thickening fraction (TF) was lower in both COPD (27.78±8.18 cm in right and 30.87±14.61 cm in left) and ILD (39.93±7.29 cm in right side and 42.18±13.02 cm in left) groups compared to healthy volunteers (49.64±8.55 cm in right and 46.49±15.26 cm in left), emphasizing its potential utility as a sensitive marker of diaphragmatic dysfunction.^8,18^ Furthermore, DTimax and TF were lower in COPD than in ILD, highlighting the greater degree of impairment in obstructive disease. Interestingly, electromyographic studies have shown that ILD patients exhibit greater diaphragmatic activation compared to COPD patients, while COPD patients demonstrate higher activity of accessory expiratory muscles.^19^ This finding reinforces the notion that distinct adaptive mechanisms underlie respiratory muscle dysfunction in the two disease entities. Moreover, consistent with our results, prior studies also confirmed that ILD patients have significantly lower DTimax and TF values than healthy subjects.^18,20^

Regarding diaphragmatic excursion (DEmax), our study found lower values in COPD patients (4.02±0.96 cm in right and 3.93±0.86 cm in left) compared to healthy controls (5.45±1.0 cm in right and 5.37±0.90 cm in left), a finding that aligns well with previously published literature.^21-23^ The pathophysiological basis of reduced excursion in COPD includes hyperinflation-induced diaphragmatic flattening, muscle fiber atrophy, and structural remodeling. These changes often involve a fiber-type shift toward type I oxidative fibers, which enhances fatigue resistance but reduces overall force-generating capacity.^24,25^ In ILD patients, excursion during tidal inspiration was relatively preserved, though maximal excursion (4.34±0.85 cm in right and 4.19±0.77 cm in left) was lower compared to healthy controls. This observation is consistent with the findings of Santana et al., who reported comparable tidal excursion between ILD and healthy individuals, but significant reductions during maximal effort.^26^ The reduction in excursion among ILD patients can be attributed to fibrotic restriction, reduced chest wall compliance, and impaired lung expansion.

The inclusion of healthy volunteers in our study provided an important reference for diaphragm ultrasound parameters. These normative values highlight the diagnostic and monitoring potential of ultrasonography in chronic lung diseases, particularly for detecting early diaphragmatic dysfunction and tracking disease progression.

Another important finding was that dyspnea severity correlated more strongly with spirometry indices (FEV1 and FVC) than with diaphragm ultrasound parameters. In both COPD and ILD groups, mMRC scores showed strong negative correlations with FEV1 and FVC, consistent with the established role of spirometry in reflecting ventilatory limitation. Beyond diaphragmatic mechanics, breathlessness is also influenced by ventilatory capacity, gas exchange abnormalities, peripheral muscle function, and altered central processing of respiratory effort.^27^

Our study is one of few studies that has been published in Nepalese context that compared ultrasound-derived diaphragmatic parameters across these three distinct groups. This makes our findings particularly valuable, as they provide a comparative insight into how obstructive and restrictive lung diseases differentially affect diaphragmatic mechanics in our context. Despite these strengths, our study has several limitations. The sample size was relatively small, which may have limited the ability to detect subtle associations, particularly between ultrasound parameters and clinical outcomes. Additionally, diaphragm ultrasonography is inherently operator-dependent, and variability in measurement technique may have introduced some degree of bias. Future studies with larger patient populations, standardized ultrasound protocols, and longitudinal follow-up are required to validate these observations and further elucidate the prognostic implications of diaphragmatic dysfunction in chronic respiratory diseases.

## CONCLUSIONS

The diaphragm thickness during tidal breathing was similar across COPD, ILD and healthy controls whereas maximal inspiratory thickness (DTimax), thickening fraction (TF), and diaphragmatic excursion (DE) were significantly reduced in COPD and ILD patients compared with healthy controls, with COPD patients showing the most pronounced impairment. Our findings are consistent with the recent evidence supporting the role of point-of-care USG of diaphragm in respiratory disease management.

## Data Availability

The data are available from the corresponding author upon reasonable request.
